# Primary Cesarean Section in Multigravida Women: Trends and Outcomes in a Tertiary Care Setting

**DOI:** 10.7759/cureus.85517

**Published:** 2025-06-07

**Authors:** Khushpreet Kaur, Sonal Srivastava Garg, Meenali Garg, Seema Grover

**Affiliations:** 1 Obstetrics and Gynaecology, Guru Gobind Singh Medical College and Hospital, Faridkot, IND

**Keywords:** incidence, maternal outcomes, multigravida, neonatal outcomes, primary cesarean

## Abstract

Introduction: A primary cesarean in a multigravida refers to the first cesarean section (CS) performed on a woman who has previously delivered vaginally at least once. This study aimed to assess the incidence and indications of primary CS in multigravida women and their feto-maternal outcomes.

Methodology: This study was conducted in the Department of Obstetrics and Gynaecology, Guru Gobind Singh Medical College and Hospital, Faridkot, Punjab, India, from June 2022 to May 2024. A total of 5,280 women delivered in our department during this period, of whom 476 multiparous antenatal women undergoing primary CS were evaluated.

Results: The incidence of primary CS in multigravida women was 39.47%, most of which were emergency CS. The most common indication for CS was fetal distress (26.47%), followed by malpresentation in 21.43% of women. Among the feto-maternal complications, 21.85% of women developed complications such as transfusion reaction (12.18%), upper respiratory tract infection (URTI) in 8.19%, puerperal pyrexia in 5.46%, and postpartum hemorrhage in 4.20% of women. Respiratory distress syndrome was the most common cause of neonatal morbidity (6.93%).

Conclusion: Multiparity increases the risk of complications in the antepartum and intrapartum periods, necessitating operative interventions in certain conditions. Efficient antenatal evaluation and early identification and management of high-risk women can help decrease the incidence of unwarranted complications leading to emergency cesareans in multigravida patients. Additionally, regular audits of the same can help optimize the incidence of CS in these women.

## Introduction

The cesarean section (CS) is one of the most common surgical procedures in obstetrics. It serves as a lifesaving intervention for both the mother and the fetus in challenging obstetric situations [1]. The rapid rise in CS rates has become a global concern, and studies conducted across India also show a concerning increase in the frequency of these deliveries [2]. In multiparous women, primary cesarean refers to the first CS performed on a woman who has previously delivered vaginally at least once [3].

For multiparous women, the need for a CS is often influenced by factors related to the infant or placenta. Nonetheless, the frequency of primary CS at teaching hospitals is increasing due to technological advancements, such as ultrasound for detecting oligohydramnios and fetal cord issues, Doppler studies showing abnormal diastolic flow, infertility treatments, unusual cardiotocography (CTG) results, and elective CS requests from patients [4]. Even a multiparous woman who has previously delivered a full-term baby vaginally can still experience cephalopelvic disproportion. As the fetus tends to grow larger with each pregnancy, it is important to accurately estimate the size of the fetus and its head. In multiparous patients, factors such as a pendulous abdomen, lumbar lordosis, and late engagement of the fetal head can increase the likelihood of malpresentations [4].

Multiparous women are at higher risk for a range of complications during both the prenatal period (such as abortion, preeclampsia, antepartum hemorrhage, multiple pregnancies, and polyhydramnios) and the intrapartum period (including malpresentation, cephalopelvic disproportion, uterine inertia, uterine rupture, retained placenta, and postpartum hemorrhage) [5]. These issues can be indicators for a CS, so careful evaluation is necessary for multiparous women. In India, most patients live in rural areas and are often referred to maternity centers only when complications arise during pregnancy or labor. There is a common belief that if a mother has had one normal delivery, subsequent births will also proceed normally, which can lead multiparous women to skip regular pregnancy check-ups.

Although a CS is a lifesaving procedure, it poses both short- and long-term health risks for both the mother and infant, potentially impacting the course and outcome of future pregnancies. Key obstetric issues associated with CS include postpartum infections, need for subsequent cesarean deliveries, uterine rupture, injuries to the bladder and bowel, abnormal placentation, and morbidly adherent placenta. Additionally, evidence suggests that CS may influence hormonal and microbiological physiology, potentially disrupting the gut flora [5]. Therefore, this study was conducted to examine the incidence and indications of primary CS in multiparous women and the feto-maternal outcomes in them. This information is essential for introspection and provides a reference line for upcoming strategies against this rapidly growing issue.

## Materials and methods

Aims and objectives

The primary aim of our study was to calculate the incidence of primary CS in multiparous women. The secondary objective was to study the indications for these cesareans and to evaluate maternal and fetal outcomes in primary CS among multiparous women.

Methods

This was a hospital-based retrospective study conducted in the Department of Obstetrics and Gynaecology, Guru Gobind Singh Medical College and Hospital, Faridkot, Punjab, India, from June 2022 to May 2024. All multiparous patients with a gestational age greater than 28 weeks who had a prior vaginal delivery were included in the study. Multiparous women with a gestational age less than 28 weeks, a scarred uterus, or a history of previous abortions only were excluded from the study.

The statistical formula used for sample size calculation is as follows:

$\text{Sample size}\text{ }(N)=\frac{Z^{2}_{(1-a/2)} p(1-p)}{d^{2}}$

where *N* is the population size, *p* is the expected prevalence (assumed to be 50% for the maximum sample size), *d* is the desired precision (5%), and *Z*^2^_(1-*a*/2) _is the critical value for a 95% confidence level (approximately 1.96).

This calculation yielded a minimum sample size of 384. Accounting for a potential 10% non-response rate, we aimed to recruit a minimum of 412 participants. However, we evaluated a total of 476 multiparous antenatal women who fulfilled our inclusion and exclusion criteria.

Methodology

All patients fulfilling the inclusion and exclusion criteria were enrolled in the study. Data pertaining to demographics, medical history, present and past obstetric history, physical examination, investigations, treatment course, indication for CS, and fetal and maternal outcomes were recorded in a self-structured proforma. The collected data were compiled into pre-validated proformas and stored in an Excel spreadsheet (Microsoft Corporation, Redmond, Washington). We used IBM SPSS Statistics for Windows, Version 21 (Released 2012; IBM Corp., Armonk, New York) as our data analysis tool.

## Results

This study of primary CS in multigravida women was carried out on those admitted to the labor room of GGSMCH, Faridkot, during a period of two years from June 2022 to May 2024. A total of 5,280 women delivered in our labor room during the study period, of whom 2,864 women (54.2%) underwent CS and 2,416 women (45.8%) delivered vaginally (Table 1). Among the 2,864 women who underwent CS, 1,144 women (39.95%) were primigravida and 1,726 women (60.05%) were multigravida. Primary cesarean was performed in 476 multigravida women (19.4%), and repeat CS was done in 1,250 multigravida women (50.9%). Thus, out of a total of 1,206 multigravida patients with previous normal deliveries, 476 underwent primary CS while the rest delivered vaginally, resulting in a 39.47% incidence rate of primary CS.

**Table 1:** Distribution of patients as per mode of delivery The data are presented as n (%).

Mode of delivery	No. of patients (N = 5,280)	
	Primigravida	Multigravida
Total patients delivered	2,824 (53.5%)	2,456 (46.5%)
Vaginal delivery (n = 2,416, 45.8%)	1,680 (59.5%)	730 (29.7%)
Cesarean section (n = 2,864, 54.2%)		
Primary CS	1,144 (40.5%)	476 (19.4%)
Repeat CS	—	1,250 (50.9%)

Out of 476 patients, the majority, that is, 350 women, were unbooked (73.94%) and only 126 patients were booked (26%) (Table 2). Among these, 305 patients (64%) were referred from smaller districts and villages. One hundred eighty-two women (38.23%) were in the age group of 25-29 years, followed by 126 (26.47%) in the age group of 20-24 years (Table 2). Ninety-six women (20.17%) were in the age group of 30-34 years, and 54 women (11.34%) were in the age group of 35-39 years. Only 12 women (2.52%) were above 40 years, and 6 patients (1.126%) were below 20 years of age.

**Table 2:** Demographic distribution of patients

Patient parameter	Category	No. of patients (N = 476)	Percentage (%)
Booking status	Unbooked	350	73.94%
	Booked	126	26.06%
Socioeconomic status	Upper class	04	0.85%
	Middle class	136	28.57%
	Lower class	336	70.50%
Age	<20 years	06	1.26%
	20–29 years	308	64.70%
	30–39 years	150	35.51%
	40–45 years	12	2.52%
Gestational age	<37 weeks	208	43.69%
	37–40 weeks	228	47.90%
	>40 weeks	40	8.40%
Parity	1	286	60.08%
	2	133	27.94%
	3	29	6.09%
	4	16	3.36%
	>4	12	2.52%

The period of gestation in 228 women (47.90%) was between 37-40 weeks, followed by 28-36+6 weeks in 208 patients (43.69%) and more than 40 weeks in 40 patients (8.40%). As per the modified Kuppuswamy Scale for socio-economic distribution, the majority of women belonged to the lower class (n = 336, 70.5%), followed by 136 (28.5%) women in the middle class, and only 4 (0.8%) patients from the upper class (Table 2).

Of the 476 cases, 411 (86.34%) underwent emergency CS, whereas only 65 (13.65 %) women were operated on electively (Table 3).

**Table 3:** Distribution of patients according to the type of lower segment cesarean section

Type of cesarean	No. of patients (N = 476)	Percentage (%)
Emergency	411	86.34%
Elective	65	13.65%

**Figure 1:**
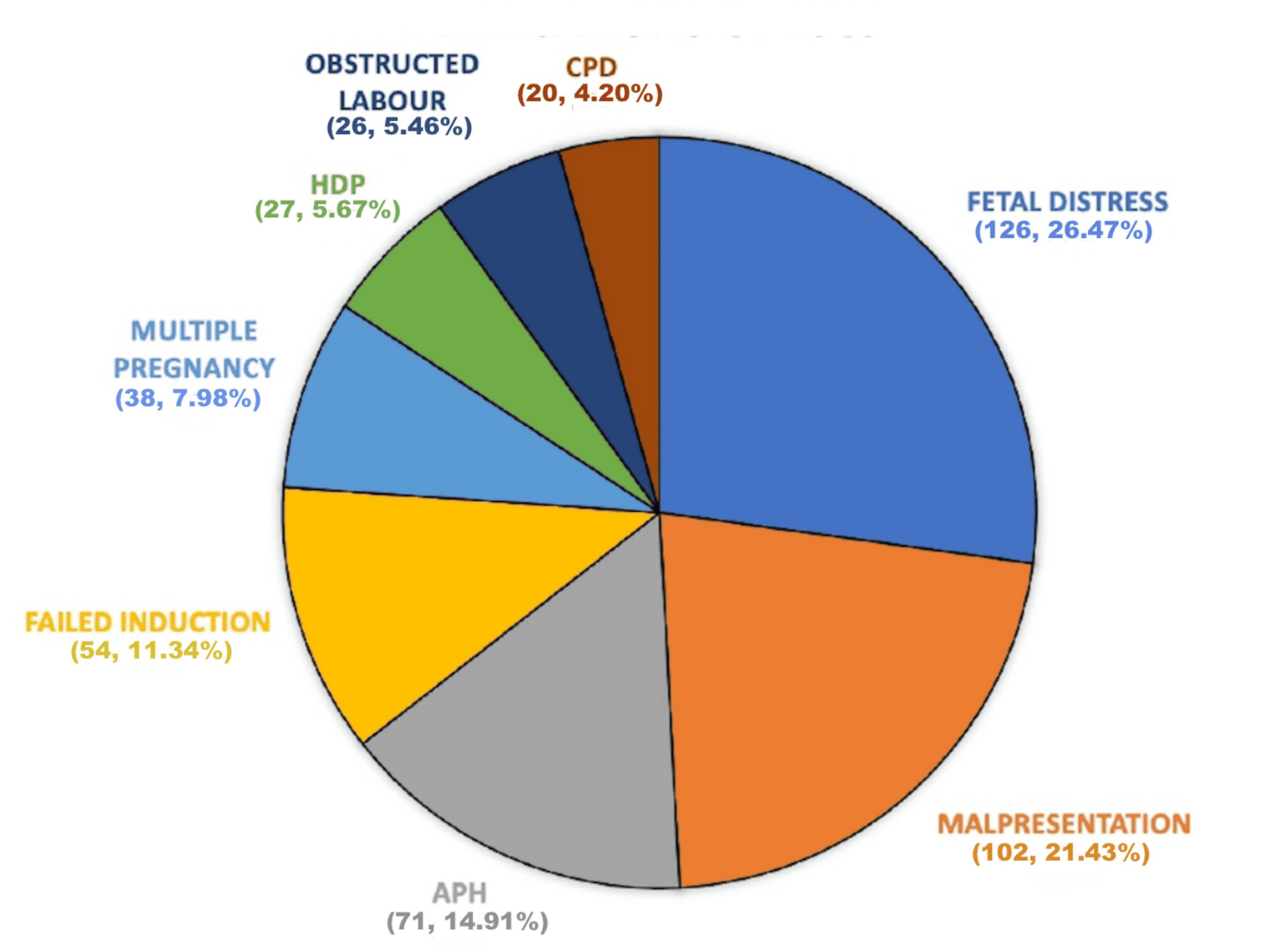
Distribution of patients according to indication of LSCS (n=476) LSCS: lower segment cesarean section, CPD: cephalopelvic disproportion, HDP: hypertensive disorders of pregnancy, APH: antepartum hemorrhage.

Fetal distress was the most common indication for CS in our study (n = 126, 26.47%), followed by malpresentation in 102 (21.43%) women, APH in 71 (14.91%) women, failed induction in 54 (11.34%), multiple pregnancy in 38 (7.98%) women, hypertensive disorders of pregnancy including eclampsia in 27 (5.67%), obstructed labor in 26 (5.46%) women, cephalopelvic disproportion in 20 (4.20%) women, and severe oligohydramnios or anhydramnios in 10 (2.10%) women (Figure 1).

**Figure 2:**
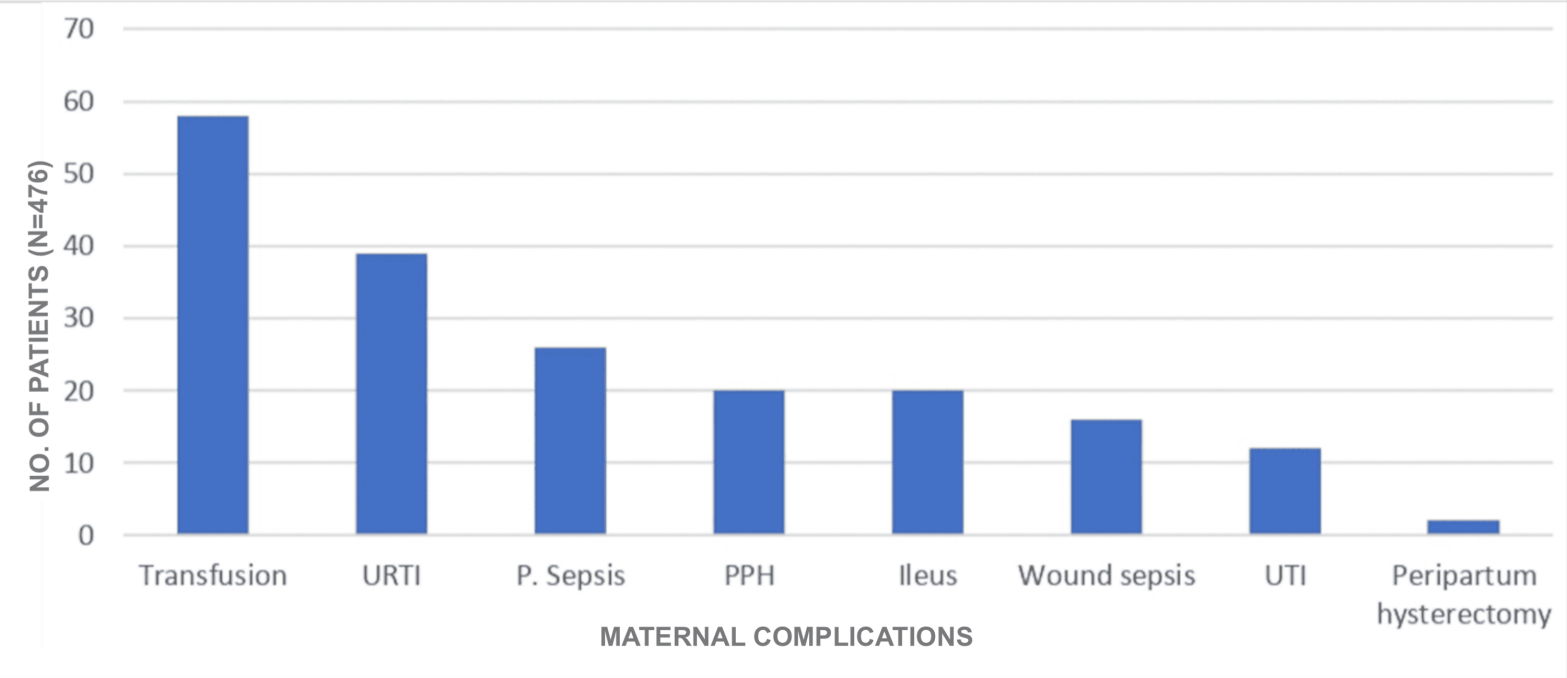
Maternal complications URTI: upper respiratory tract infection; P. Sepsis: puerperal sepsis; PPH: postpartum hemorrhage; UTI: urinary tract infection.

The majority of the women, 372 (78.15%), did not have any complications. Out of 476 patients, 58 (12.18%) women received blood transfusions. The next most common complication was upper respiratory tract infection in 39 (8.19%) women, followed by puerperal pyrexia in 26 (5.46%) women, postpartum hemorrhage in 20 (4.20%), paralytic ileus in 20 (4.20%) women, wound sepsis in 16 (3.36%) women, and peripartum hysterectomy in two women (Figure 2).

The incidence of complications according to the booking status of the women was also analyzed (Table 4). Nearly 77% of the complications were observed in unbooked patients and 23% in booked patients.

**Table 4:** Distribution of complications according to booking status The data are presented as n (%). URTI: upper respiratory tract infection, P. sepsis: puerperal sepsis, PPH: postpartum hemorrhage, UTI: urinary tract infection.

Maternal complication	Booked (22.8%)	Unbooked (77.2%)
Blood transfusion	10 (5.18%)	48 (24.8%)
URTI	8 (4.14%)	31 (16.06%)
P. sepsis	5 (2.59%)	21 (10.8%)
PPH	7 (3.62%)	13 (6.73%)
Ileus	10 (5.18%)	10 (5.18%)
Wound sepsis	2 (1.03%)	14 (7.25%)
UTI	2 (1.03%)	10 (5.18%)
Peripartum hysterectomy	0 (0%)	2 (1.03%)
Total	44 (22.8%)	149 (77.2%)

Table 5 lists the distribution of maternal complications according to the indication for primary cesareans. Women undergoing cesarean for obstructed labor developed the highest number of maternal complications, followed by those operated for antepartum hemorrhage.

**Table 5:** Distribution of maternal complications according to indication of cesarean BT: blood transfusion, URTI: upper respiratory tract infection, P. sepsis: puerperal sepsis, PPH: postpartum hemorrhage, UTI: urinary tract infection, HDP: hypertensive disorders of pregnancy, APH: antepartum hemorrhage, CPD: cephalopelvic disproportion.

Maternal complication	Foetal distress	HDP	Malpresentation	APH	Failed induction	Multiple pregnancy	Obstructed labor	CPD	Total
BT	2	6	3	15	3	10	14	5	58
URTI	1	16	2	7	1	4	6	2	39
P. Sepsis	1	3	1	5	4	3	8	1	26
PPH	-	3	1	6	2	2	6	-	20
Ileus	-	2	1	1	5	4	7	-	20
Wound sepsis	-	2	-	2	2	2	8	-	16
UTI	-	2	1	3	2	1	4	-	12
Peripartum hysterectomy	-	-	-	1	-	-	1	-	2

In our study, 438 were singleton pregnancies and 38 were multiple pregnancies (n = 514). Around 49% of the babies (n = 252) were in the weight range of 2.5-3.5 kg, whereas 45.53%, i.e., 234 babies, weighed 1.5-2.5 kg. Only 18 babies (3.50%) weighed below 1.5 kg, and 10 (1.95%) babies weighed above 3 kg (Table 6).

**Table 6:** Distribution of neonates according to birth weight

Birth weight (kg)	No. of neonates (n=514)	Percentage (%)
<1.5 kg	18	3.50%
>1.5-2.5 kg	234	45.53%
>2.5-3.5 kg	252	49.02%
>3.5-4.5 kg	08	1.56%
>4.5 kg	02	0.39%

**Figure 3:**
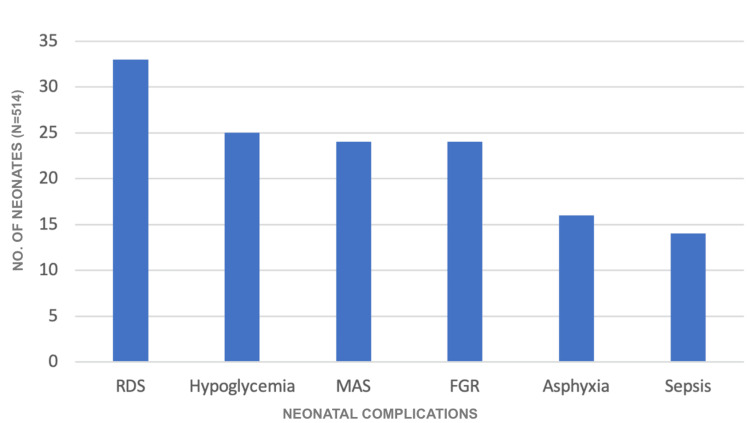
Neonatal complications RDS: respiratory distress syndrome; MAS: meconium aspiration syndrome; FGR: fetal growth restriction.

Most of the neonates, i.e., 340 (66.15%), were healthy and did not require NICU admission. The most common reason for neonatal morbidity was respiratory distress syndrome, present in 33 neonates (6.42%), followed by hypoglycemia in 25 (4.86%). Meconium aspiration syndrome and fetal growth restriction as causes of neonatal morbidity were present in 24 neonates each (4.67%) (Figure 3). Birth asphyxia was present in 16 neonates, and 14 neonates presented with neonatal sepsis. 

## Discussion

A total of 476 multigravida women undergoing primary CS were included in our study, following the application of specific inclusion and exclusion criteria. In multiparous women who have previously delivered vaginally, a CS may still be necessary to ensure a safe delivery in subsequent pregnancies. Out of the 476 participants, 350 (73.94%) had not received prior antenatal care. These findings are in line with those reported by Desai et al. (72.09%), Vijayshree et al. (68%), Kuntal et al. (78%), Rajput et al. (77.72%), and Himabindu et al. (71%) [3,6-9]. The high proportion of unbooked cases in our study may be linked to lower levels of education and socioeconomic status. In fact, 90.1% of the patients in our sample were from the lower socioeconomic class.

In our study, the largest proportion of patients (38.23%) were aged between 25 and 29 years, followed by 26.47% in the 20-24 age group. These findings are consistent with those of Vijayshree et al., where 42% of patients fell within the 25-29 age range and 30% were between 20-24 years [6]. Similarly, Rajput et al. reported that 55.95% of participants were aged 26-30 years, with 31.86% in the 21-25 year bracket [8]. Comparable age distributions were also observed in studies by Sethi P et al. [10] and Unnikrishnan B et al. [11]. Regarding gestational age, 228 women (47.90%) were between 37 and 40 weeks pregnant, while 208 women (43.69%) were between 28 and 36 weeks + 6 days. A smaller proportion, 40 women (8.40%), were beyond 40 weeks of gestation. Rajput et al. found similar trends, with 59.33% of patients in the 37-40 week range, followed by 28.76% between 32 and 36 weeks [8]. Rowaily et al. [12] also reported that the majority (78.8%) of cases were within 37-42 weeks of gestation, while 18.2% were under 37 weeks.

During the two-year study period, a total of 3,425 CS were performed, out of which 476 were primary CS in multigravida women. This represents 13.89% of all cesarean deliveries. Our findings are in alignment with those reported by Singh et al. [2], Vijayshree et al. [6], Kuntal et al. [7], and Rajput [8], who observed incidence rates of 14%, 12.61%, 14.7%, and 12.60%, respectively. Among the 476 cases, 411 patients (86.34%) underwent emergency CS, while only 65 patients (13.65%) had elective CS. These results are comparable to those from Vijayshree et al. [6], where 88% of the cases were emergency cesareans and 12% were elective. Similarly, in the study by Rajput et al. [8], 95.85% of patients underwent emergency CS, with only 4.15% having elective surgeries.

In our study, the leading indication for CS was fetal distress, observed in 126 patients (26.47%). This was followed by malpresentation in 102 cases (21.43%), antepartum hemorrhage (APH) in 71 cases (14.91%), and failed induction in 54 cases (11.34%). Other reasons included multiple pregnancies (7.98%), hypertensive disorders including eclampsia (5.67%), obstructed labor (5.46%), cephalopelvic disproportion (4.20%), and severe oligohydramnios or anhydramnios (2.10%). These findings are consistent with those of Vijayasree et al. [6] and Himabindu et al. [9], who also reported fetal distress as the most frequent cause of lower segment CS (28% and 24.7%, respectively). In contrast, Rajput et al. [8] identified malpresentation as the most common indication (29.79%), followed by fetal distress and APH, each accounting for 18.39% of cases.

In our study, the majority of patients (78.15%) did not experience any postoperative complications. This is comparable to findings by Rajput et al. [8], where 76.42% of women remained free of complications after surgery. However, a contrasting outcome was reported by Vijayasree et al. [6], where only 38% of patients had no postoperative issues. Among the 476 participants in our study, 58 patients (12.18%) required blood transfusion. The next most frequently observed complication was puerperal pyrexia in 49 cases (10.29%), followed by paralytic ileus in 42 patients (8.82%), upper respiratory tract infections in 39 cases (8.19%), wound infections in 28 (5.88%), and postpartum hemorrhage in 26 cases (5.46%), with two patients requiring peripartum hysterectomy. In the study by Vijayasree et al. [6], puerperal pyrexia was also the most common complication (14%), followed by atonic postpartum hemorrhage (12%). Blood transfusions were necessary in 10% of their cases, while wound sepsis occurred in 4%. Notably, no maternal deaths occurred in our study. This could be attributed to timely medical intervention, improved antibiotic use, access to blood transfusion, safer anesthesia practices, advanced surgical methods, and the enhanced skills of attending obstetricians.

An analysis of birth weights in our study revealed that 49.02% of newborns weighed between 2.5 and 3.5 kg, while 49.03% had a birth weight of less than 2.5 kg. Only 3.50% of infants weighed under 1.5 kg, and 1.95% were above 3 kg. In comparison, Rajput et al. [8] found that 58.29% of babies were within the 2.5-3.5 kg range, 34.97% weighed under 2.5 kg, and just 1.81% (7 babies) had a birth weight exceeding 3.5 kg. Similarly, Rowaily et al. [12] reported that the majority of neonates (61.7%) had a normal birth weight of 2.5-3.5 kg, followed by 21.6% weighing more than 3.5 kg. The lower birth weights observed in our study may be indicative of inadequate maternal nutrition and suboptimal antenatal care. In terms of neonatal outcomes, 66.15% of newborns were healthy at birth, while 33.85% required admission to the neonatal intensive care unit (NICU). The most frequently encountered cause of neonatal morbidity was respiratory distress syndrome, affecting 6.42% of neonates, followed by hypoglycemia in 4.86%. Meconium aspiration syndrome and fetal growth restriction each accounted for 4.67% of cases. Birth asphyxia was seen in 3.11% of neonates, and neonatal sepsis occurred in 2.72%.

In the study conducted by Vijayasree et al. [6], 24% of neonates required NICU admission. The leading cause of neonatal morbidity was meconium aspiration syndrome (MAS), affecting 8% of cases, followed by neonatal hypoglycemia (6%), birth asphyxia (4%), fetal growth restriction (4%), and neonatal sepsis (2%). Rajput et al. [8] reported birth asphyxia as the most prevalent neonatal complication, observed in 6.21% of cases. This was followed by respiratory distress syndrome (RDS) in 5.69%, neonatal sepsis and pyrexia in 3.36%, and MAS in 2.84%. Similarly, Sethi et al. [10] observed birth asphyxia and sepsis or pyrexia in 4% of neonates each, MAS and convulsions in 3%, and RDS in 3%. In our study, perinatal mortality occurred in 2.31% of neonates. The primary cause was birth asphyxia, responsible for 5 cases (45.45%), followed by RDS in 4 cases (36.36%), and one case each (9.09%) due to sepsis and MAS.

Limitations of our study

A key limitation of our study is that it was a retrospective analysis. A prospective study would offer the advantage of regular audits and ongoing analysis, which would contribute to more effective patient management. Additionally, retrospective studies may involve errors in data retrieval from medical records. To address these limitations, the authors plan to extend this research into a prospective study, allowing for the evaluation of trends in CS rates over the coming years.

## Conclusions

In our study, fetal distress was the most common indication for primary lower-segment CS in multigravida patients (26.47%), followed by malpresentations (21.43%). Increasing parity is a risk factor for increased complications during the antepartum, intrapartum, and postpartum periods. Therefore, effective antenatal assessment with early identification and management of high-risk cases can help reduce unnecessary complications and lower the need for emergency CS in multigravida women. Moreover, a comprehensive clinical evaluation prior to performing a CS and regular audits of these cases can contribute to optimizing CS rates in this group of patients.
